# Supplementary figures and images for: A Comparison of Methods to Measure Fitness in Escherichia coli
Source: PLoS One. 2015 May 11;10(5):e0126210. doi: 10.1371/journal.pone.0126210 (PMC4427439; doi:10.1371/journal.pone.0126210)

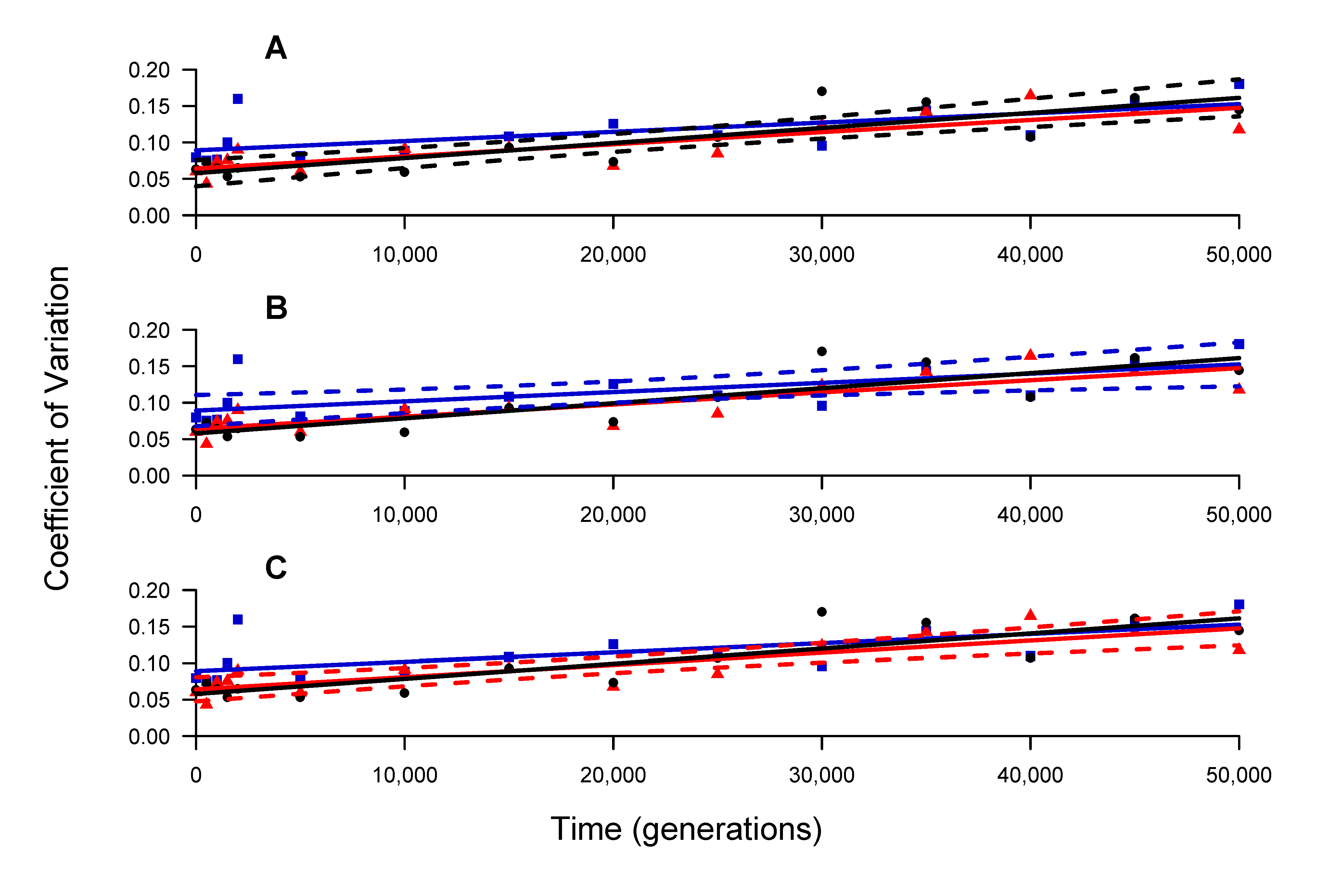

Supplement: S1 Fig — Temporal trends in the coefficient of variation across replicate assays for the three different methods used to measure fitness. Black circles show the Traditional method; blue squares show the ASR method; red triangles show the DCC method. The solid colored lines show the linear regressions based on the corresponding data. The dashed colored curves show the 95% confidence bands for the regressions for the three methods: A) Traditional, B) ASR, and C) DCC. The points and regression lines are the same across all three panels, but the confidence bands are shown separately for clarity. (TIF) [file pone.0126210.s001.tif]
