# Supplementary material for: A Comparison of Methods to Measure Fitness in Escherichia coli
Source: PLoS One. 2015 May 11;10(5):e0126210. doi: 10.1371/journal.pone.0126210 (PMC4427439; doi:10.1371/journal.pone.0126210)
Supplement: S1 Table — Analyses of variance of measured fitness values for the three methods, analyzed separately for the various generations examined. (DOC) [file pone.0126210.s002.doc]

Table S1:

|  |  | df | SS | MS | F | p |
| --- | --- | --- | --- | --- | --- | --- |
| Generation 0 | Method | 2 | 0.00452 | 0.00226 | 0.488 | 0.6219 |
|  | Block | 9 | 0.03757 | 0.00417 | 0.901 | 0.5441 |
|  | Residuals | 18 | 0.08338 | 0.00463 |  |  |
| Generation 500 | Method | 2 | 0.00097 | 0.00049 | 0.090 | 0.9147 |
|  | Block | 9 | 0.04394 | 0.00488 | 0.900 | 0.5454 |
|  | Residuals | 18 | 0.09770 | 0.00543 |  |  |
| Generation 1,000 | Method | 2 | 0.00377 | 0.00188 | 0.254 | 0.7787 |
|  | Block | 9 | 0.06030 | 0.00670 | 0.902 | 0.5437 |
|  | Residuals | 18 | 0.13375 | 0.00743 |  |  |
| Generation 1,500 | Method | 2 | 0.01654 | 0.00827 | 0.931 | 0.4123 |
|  | Block | 9 | 0.09422 | 0.01047 | 1.179 | 0.3646 |
|  | Residuals | 18 | 0.15989 | 0.00888 |  |  |
| Generation 2,000 | Method | 2 | 0.01747 | 0.00874 | 0.550 | 0.5863 |
|  | Block | 9 | 0.27162 | 0.03018 | 1.900 | 0.1178 |
|  | Residuals | 18 | 0.28588 | 0.01588 |  |  |
| Generation 5,000 | Method | 2 | 0.00561 | 0.00280 | 0.326 | 0.7258 |
|  | Block | 9 | 0.05552 | 0.00617 | 0.718 | 0.6866 |
|  | Residuals | 18 | 0.15460 | 0.00859 |  |  |
| Generation 10,000 | Method | 2 | 0.03815 | 0.01908 | 1.881 | 0.1812 |
|  | Block | 9 | 0.18114 | 0.02013 | 1.984 | 0.1032 |
|  | Residuals | 18 | 0.18257 | 0.01014 |  |  |
| Generation 15,000 | Method | 2 | 0.02064 | 0.01032 | 0.368 | 0.6973 |
|  | Block | 9 | 0.09212 | 0.01024 | 0.365 | 0.9374 |
|  | Residuals | 18 | 0.50501 | 0.02806 |  |  |
| Generation 20,000 | Method | 2 | 0.00341 | 0.00170 | 0.122 | 0.8858 |
|  | Block | 9 | 0.31731 | 0.35257 | 2.527 | 0.0450 |
|  | Residuals | 18 | 0.25116 | 0.01395 |  |  |
| Generation 25,000 | Method | 2 | 0.06087 | 0.03043 | 1.286 | 0.3006 |
|  | Block | 9 | 0.28899 | 0.03211 | 1.357 | 0.2772 |
|  | Residuals | 18 | 0.42593 | 0.02366 |  |  |
| Generation 30,000 | Method | 2 | 0.03426 | 0.01713 | 0.595 | 0.5623 |
|  | Block | 9 | 0.74363 | 0.08263 | 2.868 | 0.0273 |
|  | Residuals | 18 | 0.51864 | 0.02881 |  |  |
| Generation 35,000 | Method | 2 | 0.13333 | 0.06667 | 1.163 | 0.3349 |
|  | Block | 9 | 0.58220 | 0.06469 | 1.129 | 0.3929 |
|  | Residuals | 18 | 1.03167 | 0.05732 |  |  |
| Generation 40,000 | Method | 2 | 0.02934 | 0.01467 | 0.392 | 0.6813 |
|  | Block | 9 | 0.61796 | 0.06866 | 1.835 | 0.1306 |
|  | Residuals | 18 | 0.67359 | 0.03742 |  |  |
| Generation 45,000 | Method | 2 | 0.22400 | 0.11200 | 2.113 | 0.1499 |
|  | Block | 9 | 1.02081 | 0.11342 | 2.140 | 0.0811 |
|  | Residuals | 18 | 0.95425 | 0.05301 |  |  |
| Generation 50,000 | Method | 2 | 0.06732 | 0.03366 | 0.057 | 0.5764 |
|  | Block | 9 | 0.94686 | 0.10521 | 1.776 | 0.1432 |
|  | Residuals | 18 | 1.06627 | 0.05924 |  |  |
